# Supplementary material for: A single-sample workflow for joint metabolomic and proteomic analysis of clinical specimens
Source: Clin Proteomics. 2024 Jul 5;21:49. doi: 10.1186/s12014-024-09501-9 (PMC11225228; doi:10.1186/s12014-024-09501-9)
Supplement: Supplementary file 1 — Supplementary Material 1 [file 12014_2024_9501_MOESM1_ESM.pdf]

Supporting Information

A single-sample workflow for joint metabolomic and proteomic analysis of clinical specimens

Hagen M. Gegner*[1], Thomas Naake*[2], Karim Aljakouch*[3,4], Aurelien Dugourd*[5], Georg Kliewer[3,4], Torsten Müller[3,4], Dustin Schilling[5], Marc A. Schneider[6,7], Nina Kunze-Rohrbach[1], Thomas G.P. Grünewald[8,9,10,11], Rüdiger Hell[1], Julio Saez-Rodriguez[5], Wolfgang Huber[2], Gernot Poschet#[1], Jeroen Krijgsveld#[3,4]

* shared first author

# shared corresponding authors. GP: gernot.poschet@cos.uni-heidelberg.de; JK: j.krijgsveld@dkfz.de

[1] Centre for Organismal Studies (COS), Metabolomics Core Technology Platform, Heidelberg University, Im Neuenheimer Feld 360, 69120 Heidelberg, Germany.
[2] Genome Biology Unit, European Molecular Biology Laboratory (EMBL), Meyerhofstr. 1, 69117 Heidelberg, Germany.
[3] Faculty of Medicine, Heidelberg University, 69120 Heidelberg, Germany.
[4] Division Proteomics of Stem Cells and Cancer, German Cancer Research Center (DKFZ), Im Neuenheimer Feld 581, 69120 Heidelberg, Germany.
[5] Institute for Computational Biomedicine, Bioquant, Faculty of Medicine, Heidelberg University and Heidelberg University Hospital, Im Neuenheimer Feld 130, 69120 Heidelberg, Germany.
[6] Translational Research Unit, Thoraxklinik at Heidelberg University Hospital, Röntgenstraße 1, 69126 Heidelberg.
[7] Translational Research Center Heidelberg (TLRC), Member of The German Center for Lung Research (DZL), Im Neuenheimer Feld 156, 69120 Heidelberg.
[8] Division of Translational Pediatric Sarcoma Research, German Cancer Research Center (DKFZ), German Cancer Consortium (DKTK), Im Neuenheimer Feld 280, 69120 Heidelberg, Germany.
[9] Hopp-Children's Cancer Center (KiTZ), Heidelberg, Germany.
[10] Institute of Pathology, Heidelberg University Hospital, Im Neuenheimer Feld 224, 69120 Heidelberg, Germany.
[11] National Center for Tumor Diseases (NCT), NCT Heidelberg, a partnership between DKFZ and Heidelberg University Hospital, Germany

Table of Content

Supplementary Figures S-3

Supplementary Figure S1: Overlap of extracted proteins and peptides in FFPE, cells, plasma, and serum samples. S-3

Supplementary Figure S2: Comparison of autoSP3 and MTBE-SP3. A) Explained variance (R2) between log-transformed intensities of technical replicates. S-4

Supplementary Figure S3: GRAVY and isoelectric point scores for proteins for the sets autoSP3/MTBE-SP3. .... S-5

Supplementary Figure S4: Enriched GO terms of differentially expressed proteins between autoSP3 and MTBE-SP3 extraction. FFPE (bulk, A) and fresh-frozen tissue (bulk, B), cells (C), plasma (D), and serum (E). S-6

Supplementary Figure S5: Enriched GO terms of differentially expressed proteins for the contrast TT vs. NAT in the lung adenocarcinoma dataset. S-7

Supplementary Tables S-8

Supplementary Table S1: Differentially expressed proteins between autoSP3 and MTBE-SP3 and coefficient of variation (CV) between replicates. S-8

Supplementary Table S2: Spearman ρ correlation coefficients between GRAVY scores or isoelectric point values and t-values. S-9

Reference S-10

Supplementary Tables (in Excel file)

Supplementary Table S3. MaxQuant ProteinGroups output tables for Cell, Fresh-Frozen, FFPE, Plasma, and Serum samples.

Supplementary Table S4. MaxQuant ProteinGroups output table for the lung cancer samples cohort.

Supplementary Figures

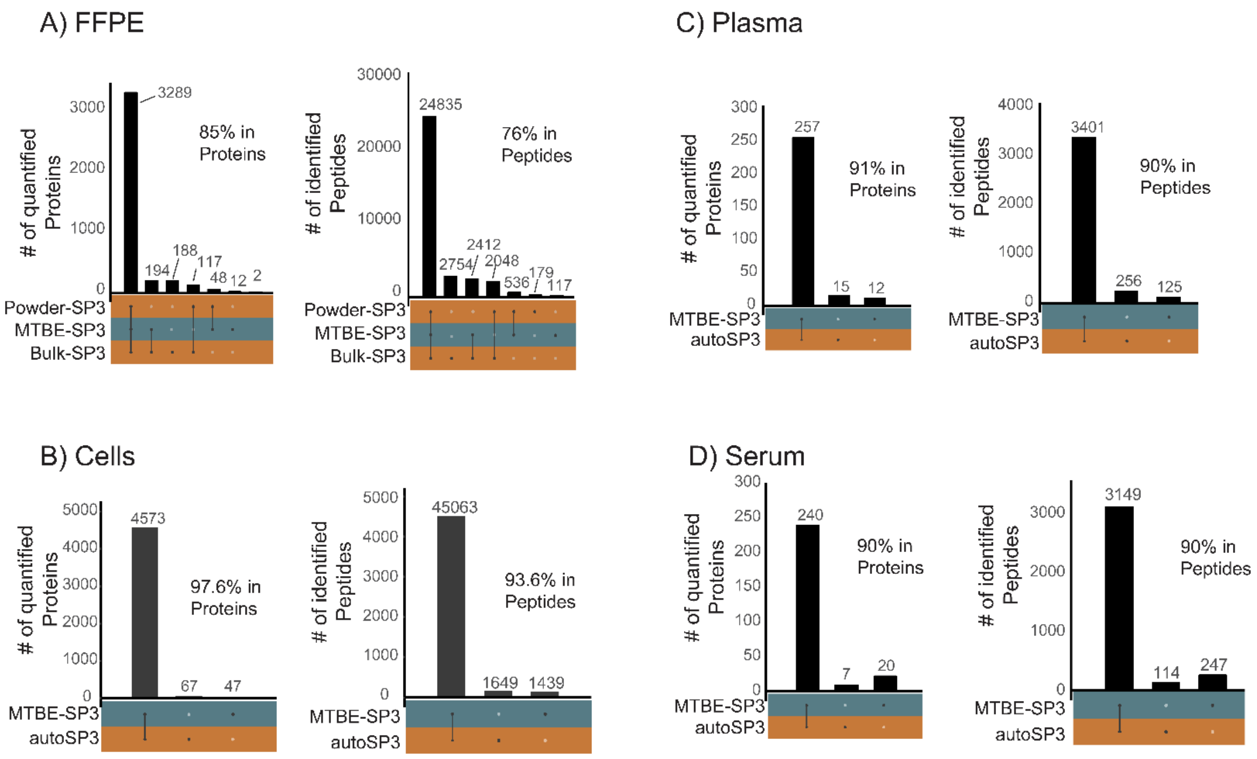

Supplementary Figure S1: Overlap of extracted proteins and peptides in FFPE, cells, plasma, and serum samples. Joint and disjoint proteins and peptide sets. A) FFPE tissue. About 85% of proteins and 76% of peptides were detected in the joint set autoSP3 (Powder, Bulk) and MTBE-SP3. B) Cells. About 97.6% of proteins and 93.6% of peptides were detected in the joint set autoSP3 and MTBE-SP3. C) Plasma. About 91% of proteins and 90% of peptides were detected in the joint set autoSP3 and MTBE-SP3. D) Serum. About 90% of proteins and 90% of peptides were detected in the joint set autoSP3 and MTBE-SP3.

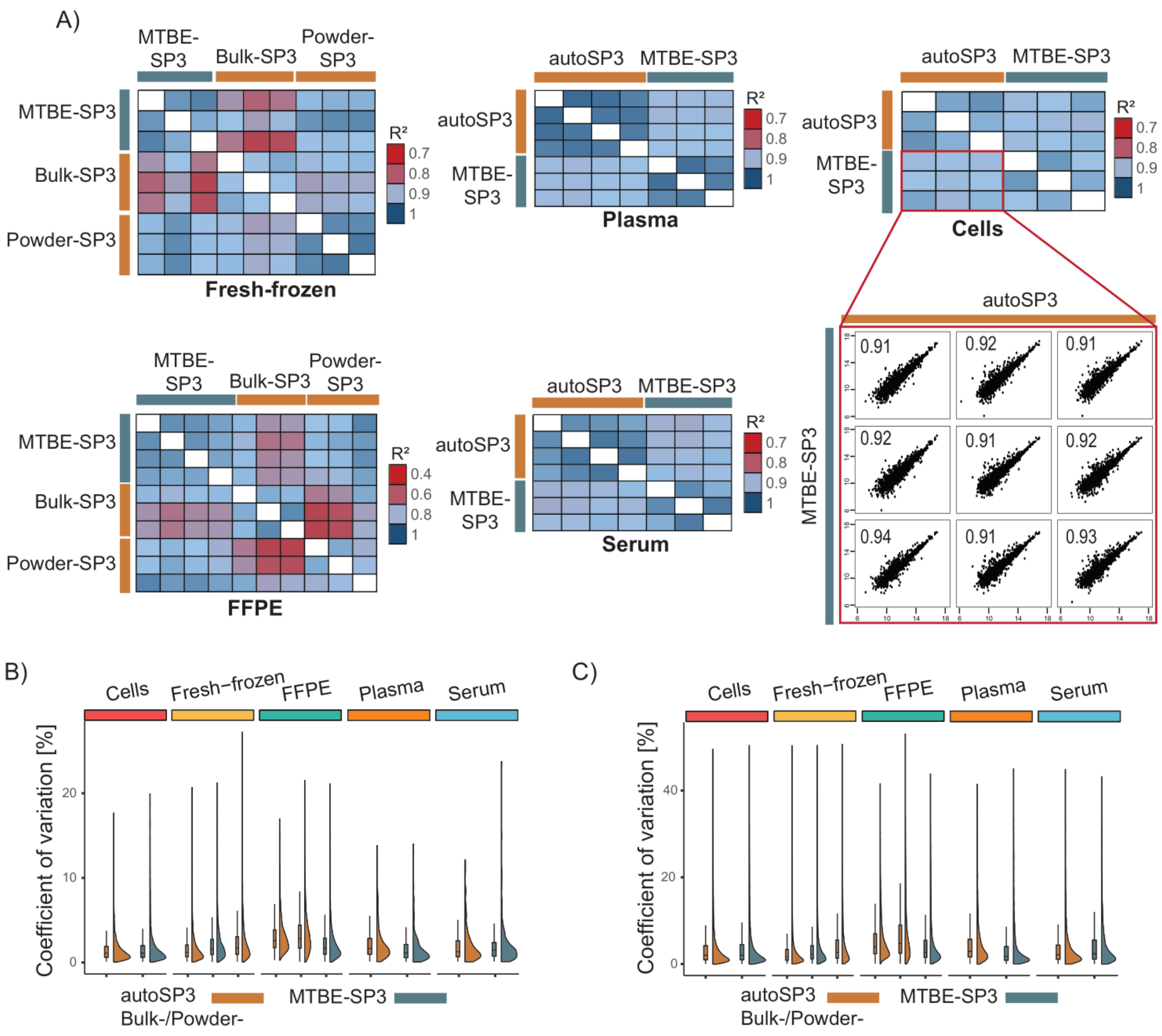

Supplementary Figure S2: Comparison of autoSP3 and MTBE-SP3. A) Explained variance (R2) between log-transformed intensities of technical replicates. autoSP3 and MTBE-SP3 show overall high R^2 between log-transformed intensities of replicates in all sample types as exemplified by the scatter plot for log-transformed intensities of autoSP3 and MTBE-SP3 in cells. The numerical values within the subpanel denotes the R^2 between autoSP3 and MTBE-SP3 technical replicates. B) CV values of log-transformed protein intensities of technical replicates for autoSP3 and MTBE-SP3. MTBE-SP3 shows CV values in a similar range to autoSP3 in all sample types. C) CV values of log-transformed peptide intensities of technical replicates for autoSP3 and MTBE-SP3. MTBE-SP3 shows CV values in a similar range to autoSP3 in all sample types. CV: coefficient of variation.

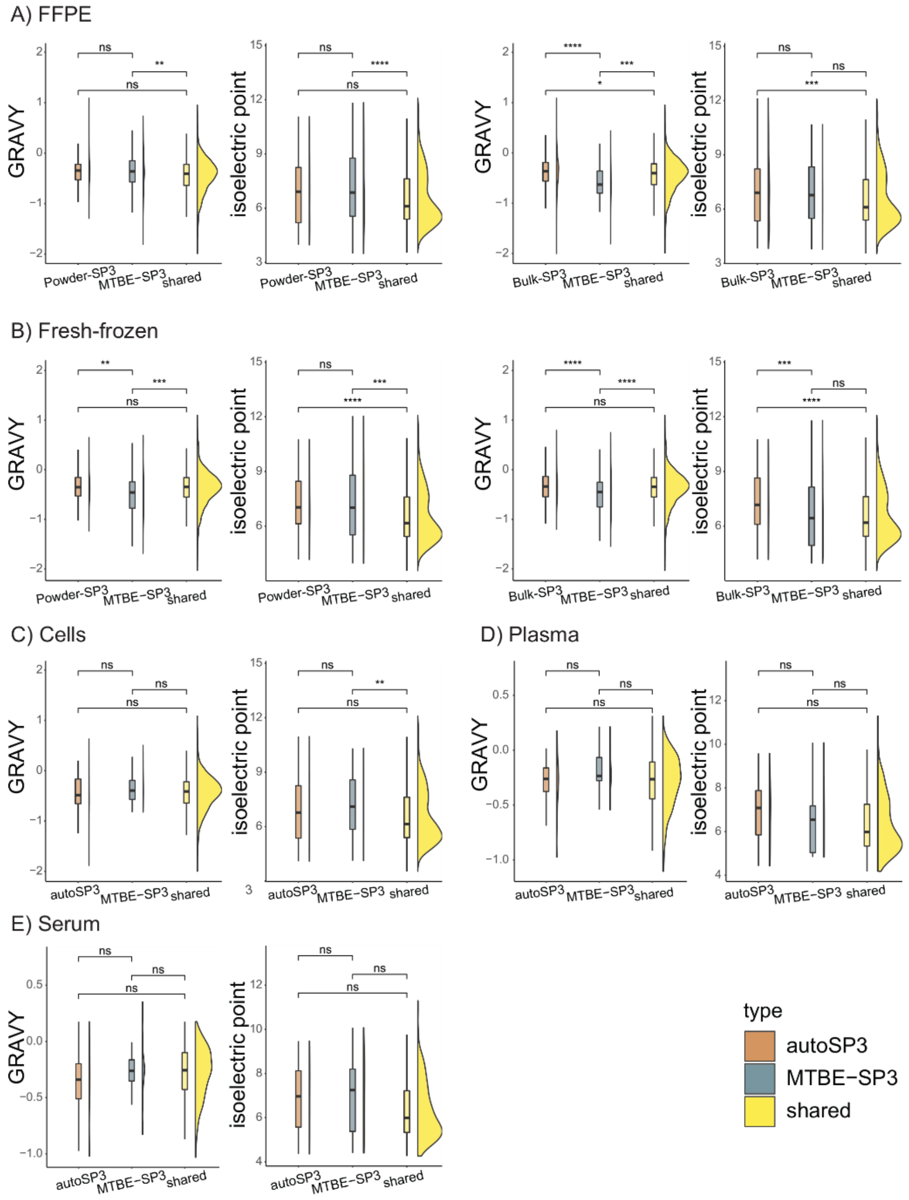

Supplementary Figure S3: GRAVY and isoelectric point scores for proteins for the sets autoSP3/MTBE-SP3. Differences in means of GRAVY and isoelectric point values between the protein sets that were unique to autoSP3/MTBE-SP3 or shared (common) between autoSP3 and MTBE-SP3 were tested by the Wilcoxon signed-rank test (no adjustment for multiple testing). The effect size of differences was generally small, but statistically significant due to the high number of proteins. A) FFPE tissue. The two subfigures on the left refer to the contrast 'Powder-SP3 *vs.* Powder-MTBE-SP3'. The two subfigures on the right refer to the contrast 'Bulk-SP3 *vs.* Powder-MTBE-SP3'. B) Fresh-frozen tissue. The two subfigures on the left refer to the contrast 'Powder-SP3 *vs.* Powder-MTBE-SP3'. The two subfigures on the right refer to the contrast 'Bulk-SP3 *vs.* Powder-MTBE-SP3'. C) Cells. D) Plasma. E) Serum. *: p-value < 0.05, **: p-value < 0.01, ***: p-value < 0.001, ****: p-value < 0.0001.

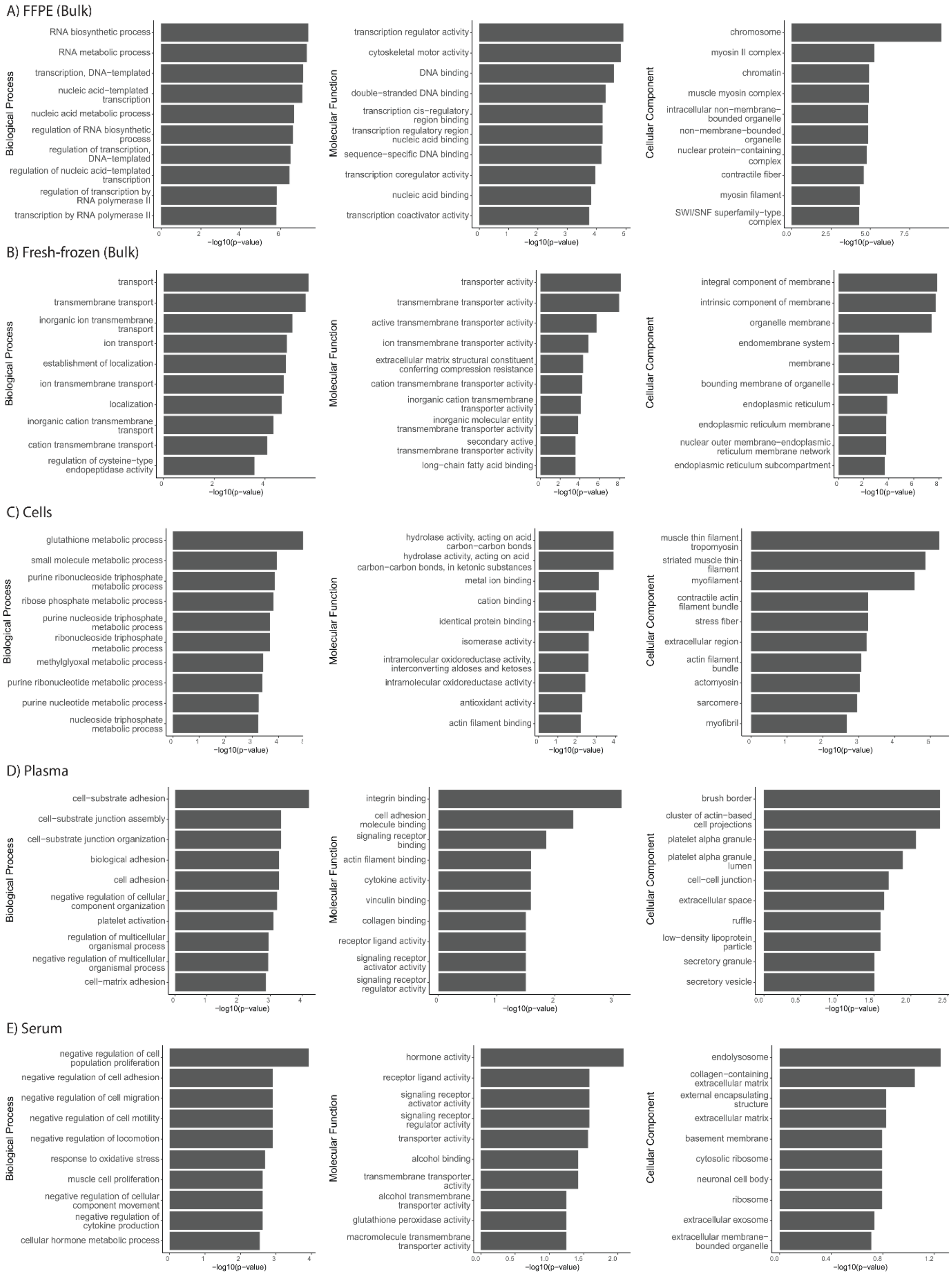

Supplementary Figure S4: Enriched GO terms of differentially expressed proteins between autoSP3 and MTBE-SP3 extraction. FFPE (bulk, A) and fresh-frozen tissue (bulk, B), cells (C), plasma (D), and serum (E). Shown are the top 10 terms for the categories Biological Process, Molecular Function, and Cellular Compartment.

A)

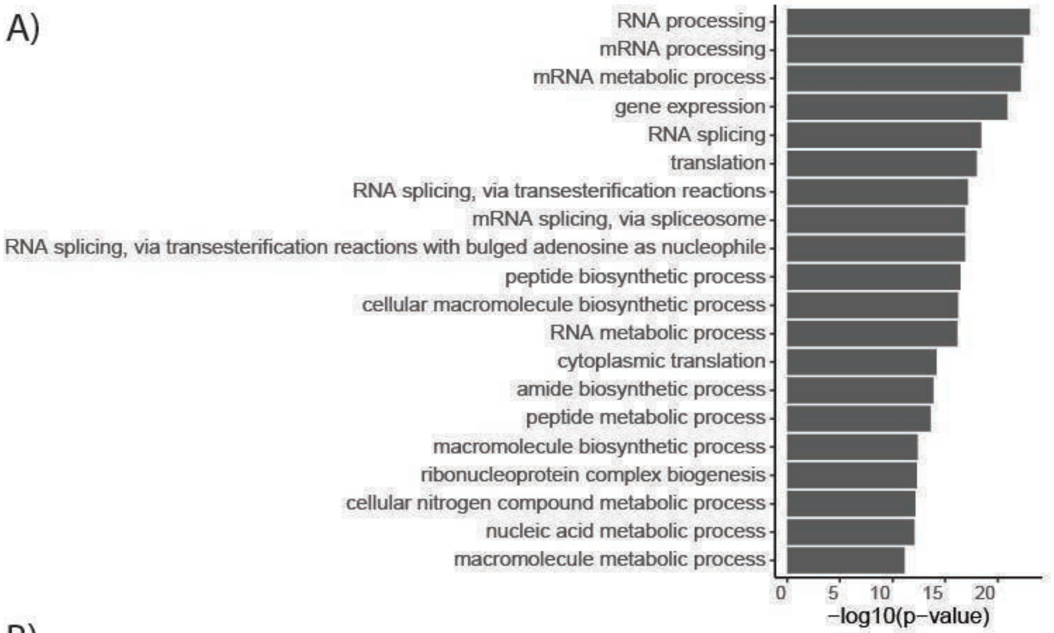

B)

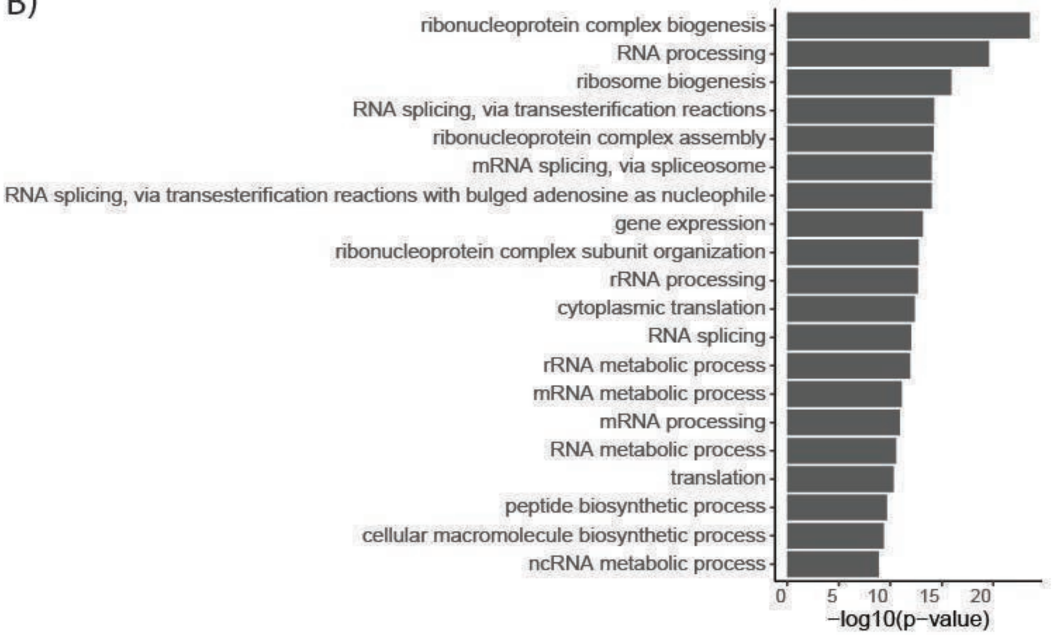

Supplementary Figure S5: Enriched GO terms of differentially expressed proteins for the contrast TT vs. NAT in the lung adenocarcinoma dataset. A) GO terms for proteomics dataset acquired using the autoSP3 extraction. B) GO terms for proteomics dataset acquired using the MTBE-SP3 extraction. Shown are the top 20 terms for the category Biological Process. NAT: non-tumorous adjacent tissue. TT: tumorous tissue.

Supplementary Tables

Supplementary Table S1: Differentially expressed proteins between autoSP3 and MTBE-SP3 and coefficient of variation (CV) between replicates. For all tissues, DE proteins were determined using linear models by testing differences between the replicates extracted with autoSP3 *vs.* the replicates extracted by MTBE-SP3. Reported here are the number of significantly DE proteins ($\alpha < 0.05$ after FDR correction) for each experiment. The number in brackets shows the total number of tested proteins. The percent of significantly DE proteins was calculated from the number of significantly DE proteins and total number of tested proteins. The CV values were calculated from the mean of and standard deviation between technical replicates of each condition, e.g. of the autoSP3-derived technical replicates and the MTBE-SP3-derived technical replicates of the cell dataset. CV values are reported in percent. CV: coefficient of variation; DE: differentially expressed.

**Sample type**	**number of significantly DE proteins**	**Percent of significantly DE proteins (in %)**	**mean of CV (in %, autoSP3)**	**mean of CV (in %, MTBE-SP3)**
FFPE tissue (Powder)	0 (3337)	0	3.3	2.4
FFPE tissue (Bulk)	527 (3483)	15.1	3.1	2.3
Fresh-frozen tissue (Powder)	0 (4096)	0	1.7	2.1
Fresh-frozen tissue (Bulk)	782 (4046)	19.3	2.4	2.1
Cells	52 (4573)	1.1	1.5	1.6
Plasma	37 (257)	14.4	2.3	1.6
Serum	11 (240)	4.6	2.0	1.9

Supplementary Table S2: Spearman ρ correlation coefficients between GRAVY scores or isoelectric point values and t-values. GRAVY scores and isoelectric point values were derived from the amino acid sequences of proteins. For each tissue, the t-values from differential expression analysis derived from the protein abundances were correlated using Spearman's Rank correlation against the GRAVY scores or isoelectric point values.

**Sample type**	**ρ (GRAVY)**	**ρ (isoelectric point)**
FFPE tissue<br>(Powder, autoSP3 *vs.* MTBE-SP3)	0.04	-0.01
FFPE tissue (Bulk, autoSP3 *vs.* MTBE-SP3)	0.26	-0.008
Fresh-frozen tissue<br>(Powder, autoSP3 *vs.* MTBE-SP3)	0.31	0.1
Fresh-frozen tissue<br>(Bulk, autoSP3 *vs.* MTBE-SP3)	0.27	0.1
Cells (autoSP3 *vs.* MTBE-SP3)	-0.001	-0.02
Plasma (autoSP3 *vs.* MTBE-SP3	0.02	0.11
Serum (autoSP3 *vs.* MTBE-SP3)	0.008	0.02

Reference

(1) Naake, T.; Huber, W. MatrixQCvis: Shiny-Based Interactive Data Quality Exploration for Omics Data. *Bioinformatics* **2022**, *38* (4), 1181–1182. https://doi.org/10.1093/BIOINFORMATICS/BTAB748.

(2) Conway, J. R.; Lex, A.; Gehlenborg, N. UpSetR: An R Package for the Visualization of Intersecting Sets and Their Properties. *Bioinformatics* **2017**, *33* (18), 2938–2940. https://doi.org/10.1093/BIOINFORMATICS/BTX364.

(3) Kozlowski, L. P. IPC - Isoelectric Point Calculator. *Biol. Direct* **2016**, *11* (1), 1–16. https://doi.org/10.1186/S13062-016-0159-9/FIGURES/4.

(4) Kyte, J.; Doolittle, R. F. A Simple Method for Displaying the Hydropathic Character of a Protein. *J. Mol. Biol.* **1982**, *157* (1), 105–132. https://doi.org/10.1016/0022-2836(82)90515-0.
